# Real-world efficacy of Bruton tyrosine kinase inhibitor based maintenance therapy in diffuse large B-cell lymphoma: a multicenter retrospective cohort study with external historical control

**DOI:** 10.1080/15384047.2026.2662031

**Published:** 2026-04-21

**Authors:** Li-Wei Lyu, Na Yao, Zi-Xian Liu, Yan-Ying Wang, Jie Li, He-Bing Zhou, Li-Hong Li, Zhen-Ling Li, Liang Wang

**Affiliations:** a Department of Hematology, Beijing Tongren Hospital, Capital Medical University, Beijing, China; b Department of Hematology, China-Japan Friendship Hospital, Beijing, China; c Department of Hematology, Beijing Tsinghua Changgung Hospital, School of Clinical Medicine, Tsinghua University, Beijing, China; d Department of Hematology, Beijing Luhe Hospital, Capital Medical University, Beijing, China

**Keywords:** Diffuse large B-cell lymphoma, maintenance therapy, survival, BTKi, central nervous system

## Abstract

**Introduction:**

The selection of an optimal maintenance agent in diffuse large B-cell lymphoma (DLBCL) continues to pose a significant clinical challenge. This study aims to evaluate the prognostic impact of maintenance therapy (MT) in DLBCL.

**Methods:**

We conducted a retrospective analysis of data from DLBCL patients undergoing first-line MT at four hospitals in Beijing between January 2019 and August 2024. The REMoDL-B trial database was selected as the control group.

**Results:**

The MT group comprised 106 cases and a median follow-up duration of 25.4 months. The rates of progression and death were 11.32% (12/106) and 1.89% (2/106), respectively. The 2-y progression-free survival (PFS) and overall survival (OS) rates were 90% and 98%, respectively. The MT group demonstrated significantly superior PFS and OS compared to the control group (*p* = 0.024, *p* = 0.008). Furthermore, multivariate analysis indicated that MT (*p* = 0.021, OR = 0.037, 95% CI, 0.002–0.605) was an independent prognostic factor associated with improved PFS. For patients receiving Bruton tyrosine kinase inhibitors (BTKi), the 2-y PFS and OS rates were 87.6% and 97.2%, respectively, both significantly better than those of the control group (*p* = 0.048, *p* = 0.024). Despite 43.6% of patients being at high risk for central nervous system (CNS), no CNS recurrences were observed. The PFS of the MCD subtype is better than that of the A53 subtype.

**Conclusions:**

While limited by the retrospective study, our analysis raises the hypothesis that MT may correlate with improved DLBCL outcomes. A similar trend suggesting potential benefit from BTKi maintenance was noted, meriting further investigation in controlled settings.

## Introduction

Diffuse large B-cell lymphoma (DLBCL) is the most prevalent subtype of non-Hodgkin lymphoma, comprising approximately 40% of all lymphoma cases worldwide.^1^ This malignancy is aggressive and exhibits considerable clinical, morphological, and molecular heterogeneity, which presents significant challenges for its treatment and management. Despite the substantial improvements in patient prognosis brought about by the introduction of rituximab,^2^ approximately 30% of patients experience relapse following initial therapy.^3^ The prognosis for relapsed patients is notably poor, with reported 1-y and 5-y survival rates at only 41% and 27%, respectively, and a median survival duration of just 10 months.^4^ Therefore, efforts to reduce relapse rates and extend progression-free survival (PFS) are essential for enhancing outcomes in DLBCL.

To tackle this challenge, intensified induction regimens have been extensively investigated. Nevertheless, such intensified chemotherapy regimens result in increased treatment-related toxicity without yielding significant improvements in survival outcomes.^5^ Consolidation strategies, including radiotherapy and autologous hematopoietic stem cell transplantation (ASCT), have also been explored but have not demonstrated substantial benefits.^5^ Moreover, these approaches are frequently inappropriate for elderly patients. At present, extending survival beyond the attainment of high remission rates remains a critical unmet need.

Maintenance therapy (MT) is recognized as a strategy aimed at sustaining the initial response achieved through induction therapy, thereby prolonging remission duration, delaying relapse, and enhancing long-term survival. This approach has been successfully applied to various hematologic malignancies, including multiple myeloma and indolent lymphomas.^6-8^ However, its clinical efficacy in DLBCL appears limited. Specifically, rituximab maintenance has not demonstrated a survival advantage in DLBCL.^9^^,^^10^ Although lenalidomide maintenance may extend PFS, particularly in elderly patients, it similarly fails to show a significant overall survival (OS) benefit.^11^ In recent years, a range of novel targeted agents, such as polatuzumab vedotin and Bruton's tyrosine kinase inhibitors (BTKi), have been developed and are increasingly being integrated into first-line DLBCL treatment regimens. The potential impact of these new agents on the existing therapeutic paradigm remains uncertain. Furthermore, there is a significant paucity of data supporting the role of MT in improving outcomes and guiding patient selection within this context. In clinical practice, we observed that the prognosis of some patients who received BTKi as maintenance therapy seemed to improve. This study aims to evaluate the impact of MT on the prognosis of DLBCL patients in the era of novel agents.

## Methods

### Study cohort

We conducted a retrospective analysis of data from patients with DLBCL who underwent first-line MT between January 2019 and August 2024 at four hospitals in Beijing: Beijing Tongren Hospital, China-Japan Friendship Hospital, Beijing Tsinghua Changgung Hospital, and Beijing Luhe Hospital. The follow-up period extended until May 30, 2025. This study adhered to the principles outlined in the Declaration of Helsinki and received approval from the Medical Ethics Committee of Beijing Tongren Hospital. Informed written consent was obtained from all participants in accordance with the Declaration of Helsinki. The inclusion criteria encompassed patients with histologically confirmed DLBCL, aged 18 y or older, who achieved either a complete response (CR) or partial response (PR) at the end of induction therapy (EOT) assessment. Exclusion criteria comprised primary central nervous system lymphoma, incomplete clinical data, voluntary discontinuation of maintenance therapy, or a switch to a different maintenance agent. Involvement of an immune-privileged site was defined as a disease affecting the central nervous system (CNS), eyes, or testes. Genomic DNA was extracted from tumor tissue samples for next-generation sequencing (NGS). Molecular subtyping was performed according to Wright et al.'s “seven-gene” genotyping model.^12^ The DLBCL patients who had not received first-line maintenance therapy in the publicly available REMoDL-B trial database were selected as the control group. The dataset had relatively complete baseline data and a balanced distribution of low-risk and high-risk patients. For REMoDL-B dataset, these follow-up data were available at GSE117556 (https://www.ncbi.nlm.nih.gov/geo/query/acc.cgi?acc=GSE117556).

### Treatment schedule and therapeutic outcomes

The initial induction therapy comprised 6 to 8 cycles of rituximab-based multi-agent combination treatment. An interim response assessment was conducted utilizing PET-CT following the completion of 4 cycles of induction therapy. The EOT response assessment was performed using PET-CT 6 to 8 weeks subsequent to the conclusion of the induction therapy. Clinical response was evaluated in accordance with the Revised International Working Group (IWG) criteria for malignant lymphoma (Lugano classification). During the maintenance phase, follow-up evaluations were scheduled at intervals of 3 to 6 months. Adverse events were assessed in alignment with the Common Terminology Criteria for Adverse Events (CTCAE) version 5.0. PFS was calculated from the initiation date of treatment to the date of confirmed disease progression or the last follow-up date, whichever occurred first. OS was calculated from the initiation date of treatment to the date of death from any cause or the last follow-up date, whichever occurred first. PFS_m_ was calculated from the initiation date of maintenance therapy to the date of confirmed disease progression or the last follow-up date, whichever occurred first. OS_m_ was calculated from the initiation date of maintenance therapy to the date of death from any cause or the last follow-up date, whichever occurred first.

### Statistical analysis

Statistical analyses were conducted utilizing IBM SPSS Statistics version 25.0. To achieve balance in baseline characteristics between the two groups, propensity score matching (PSM) was implemented. The technique uses a Logistic regression model, without replacement. This technique was employed to control for confounding variables across both groups by incorporating gender, age, Ann Arbor stage, International Prognostic Index (IPI), extranodal lesions, lactate dehydrogenase (LDH) levels, cell of origin (COO) classification, and double-expression status as covariates, with a caliper width of 0.2. Categorical variables were presented as frequencies and percentages and were analyzed using the Chi-square test or Fisher's exact test, as appropriate. Continuous variables were summarized as medians and compared using the Mann-Whitney U test. Survival analysis was performed via the Kaplan–Meier method, with survival curves generated for visualization purposes. The log-rank test was employed to compare survival curves between groups. Multivariable analysis was conducted using Cox proportional hazards regression modeling. All statistical tests were two-sided, with a threshold for statistical significance set at *p* < 0.05. Figures were produced using GraphPad Prism version 9.

## Results

### Patients characteristics

**Figure 1.**
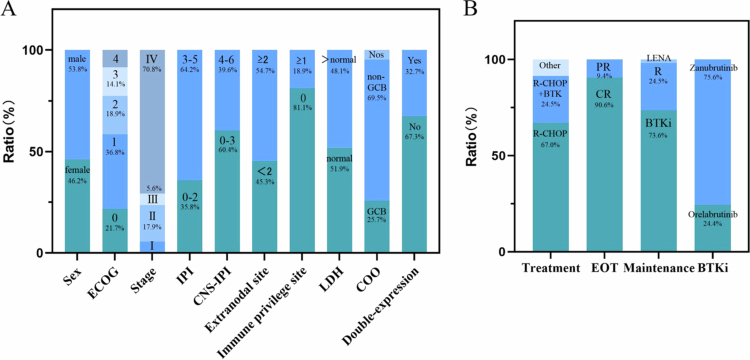
Clinical characteristics of DLBCL patients receiving maintenance therapy (*n* = 106). (A) Baseline data of patients receiving maintenance therapy. (B) Treatment data of patients receiving maintenance therapy. ECOG Eastern Cooperative Oncology Group, IPI International Prognostic Index, CNS central nervous system, LDH lactate dehydrogenase, COO cell of origin, EOT end of induction therapy, BTKi Bruton tyrosine kinase inhibitors, R rituximab, LENA lenalidomide.

This study comprised 106 participants in the MT group, with a median age of 69 y (range, 22–90 y). The clinical characteristics are illustrated in Figure 1. The MT group exhibited a higher proportion of patients over 60 y of age, Eastern Cooperative Oncology Group (ECOG) ≥ 2, stages III-IV, IPI ≥ 3, high-risk CNS-IPI group, having more than one extranodal site, involvement of immune-privileged sites, elevated LDH levels, and a non-germinal center B-cell (non-GCB) subtype. Using PSM, 44 patients with DLBCL from the database were selected to form the control group. The baseline characteristics of the MT group, post-matching with the control group, are presented in Table 1. The primary induction regimens for the MT group included R-CHOP-like regimens, R-CHOP combined with BTKi, and chemotherapy-free regimens. All patients in the control group received R-CHOP with or without bortezomib as induction regimens.

**Table 1.** Clinical characteristics of DLBCL patients after matching.

	Maintenance group (*n* = 44)	Control group (*n* = 44)	*p*-value	SMD	BTKi (*n* = 37)	Control group (*n* = 37)	*p*-value	SMD
**Sex**								
Male	22 (50.0)	27 (61.4)	0.283	0.228	18 (48.6)	25 (67.6)	0.099	0.385
Female	22 (50.0)	17 (38.6)			19 (51.4)	12 (32.4)		
**Age**								
>60	25 (56.8)	29 (65.9)	0.381	0.185	27 (73.0)	24 (64.9)	0.451	0.173
≤60	19 (43.2)	15 (34.1)			10 (27.0)	13 (35.1)		
**Ann Arbor**								
III–IV	33 (75.0)	29 (65.9)	0.350	0.198	24 (64.9)	23 (62.2)	0.809	0.055
I–II	11 (25.0)	15 (34.1)			13 (35.1)	14 (37.8)		
**IPI**								
3–5	22 (50.0)	18 (40.9)	0.392	0.181	16 (43.2)	15 (40.5)	0.814	0.054
0–2	22 (50.0)	26 (59.1)			21(56.8)	22 (59.5)		
**Extranodal lesions**								
>1	20 (45.5)	20 (45.5)	1.000	<0.001	18 (48.6)	16 (43.2)	0.641	0.107
≤1	24 (54.5)	24 (54.5)			19 (51.4)	21 (56.8)		
**LDH**								
>250U/L	17 (38.6)	17 (38.6)	1.000	<0.001	13 (35.1)	16 (43.2)	0.475	0.164
≤250U/L	27 (61.4)	27 (61.4)			24 (64.9)	21 (56.8)		
**COO**								
non-GCB	27 (61.4)	20 (45.5)	0.135	0.319	28 (75.7)	20 (54.1)	0.051	0.459
GCB	17 (38.6)	24 (54.5)			9 (24.3)	17 (45.9)		
Double-expression	*n* = 43	*n* = 14			*n* = 36	*n* = 13		
Yes	15 (34.9)	6 (42.9)	0.591	0.160	12 (33.3)	5 (38.5)	0.739	0.104
No	28 (65.1)	8 (57.1)			24 (66.7)	8 (61.5)		

IPI – international prognostic index, LDH – lactate dehydrogenase, COO – cell of origin, BTKi – Bruton tyrosine kinase inhibitors, SMD – standardized mean differences.

### Survival analysis in MT group

**Figure 2.**
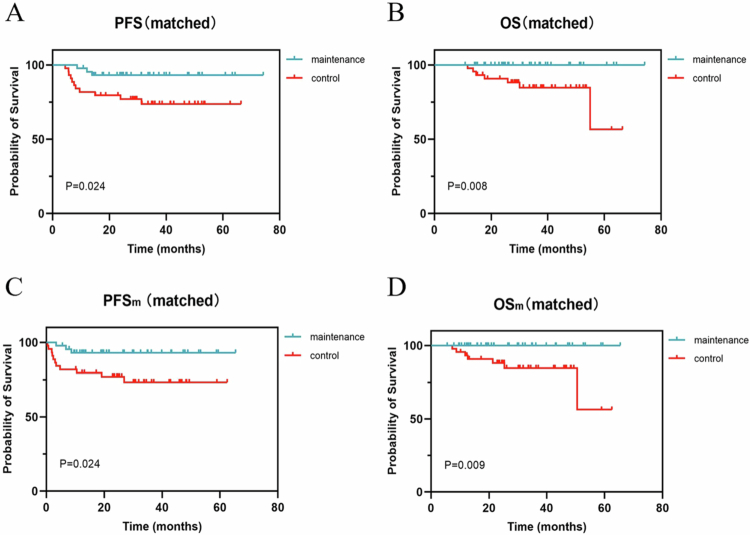
Survival analysis comparing maintenance therapy group to the REMoDL-B cohort (control group). (A and B) PFS and OS were calculated from the initiation date of the treatment. (C and D) PFS_m_ and OS_m_ were calculated from the initiation date of maintenance therapy. PFS progression-free survival, OS overall survival.

**Figure 3.**
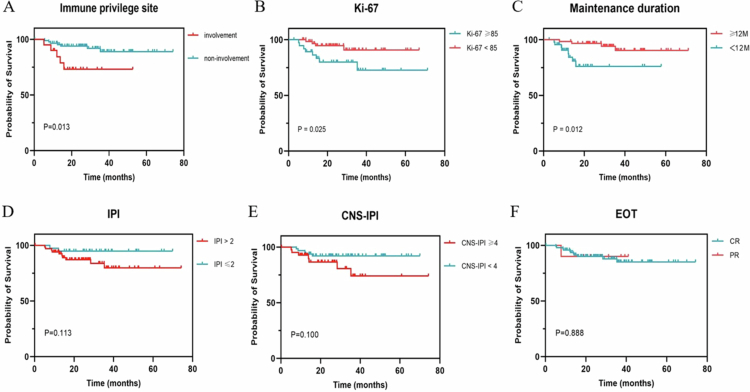
Prognostic factors for progression-free survival in maintenance therapy. The PFS survival curve of immune privilege site involvement or not (A), Ki-67 level (B), duration of maintenance therapy (C), IPI score (D), CNS-IPI score (E) and efficacy at the end of induction therapy (F). IPI International Prognostic Index, CNS central nervous system, EOT end of induction therapy.

With a median follow-up period of 25.4 months, the median PFS and OS were not reached (Figure 2). The rates of progression and death were 11.32% (12/106) and 1.89% (2/106), respectively. The 2-y PFS and OS rates were 90% and 98%, respectively. In the high-risk groups defined by the IPI (IPI score ≥ 3) and the CNS-IPI (CNS-IPI score ≥ 4), the 2-y PFS rates were 87.0% and 86.5%, respectively. Both univariate and multivariate analyses identified immune-privileged site involvement (*p* = 0.013, 0.028), Ki-67 ≥ 85% (*p* = 0.025, 0.005), and duration of maintenance (DOM) less than 12 months (*p* = 0.012, 0.044) as independent risk factors for PFS (Figure 3A–C). High-risk IPI and CNS-IPI scores did not significantly impact PFS (Figure 3D and E). No significant difference in PFS was observed between patients achieving CR versus PR (with 80% of these patients being aged 70 y or older) at the EOT (Figure 3F). In contrast, within the control group, patients achieving CR had significantly better PFS compared to those achieving PR (*p* = 0.034).

### Survival analysis between MT group and control group

The PFS and OS in the MT group were significantly superior to those in the control group (*p* = 0.024, *p* = 0.008; Figure 2A and B). The PFS_m_ and OS_m_ in the MT group were significantly superior to those in the control group (*p* = 0.024, *p* = 0.009; Figure 2C and D). Multivariate analysis of induction therapy, MT, IPI and double-expression showed that MT and IPI were independent prognostic factors for PFS (*p* = 0.021, HR = 0.037, 95% CI, 0.002–0.605; *p* = 0.026, HR = 20.526, 95% CI, 1.448–290.998), and PFS_m_ (*p* = 0.021, HR = 0.037, 95% CI, 0.002–0.609; *p* = 0.026, HR = 20.460, 95% CI, 1.445–289.637).

### BTKi maintenance

**Figure 4.**
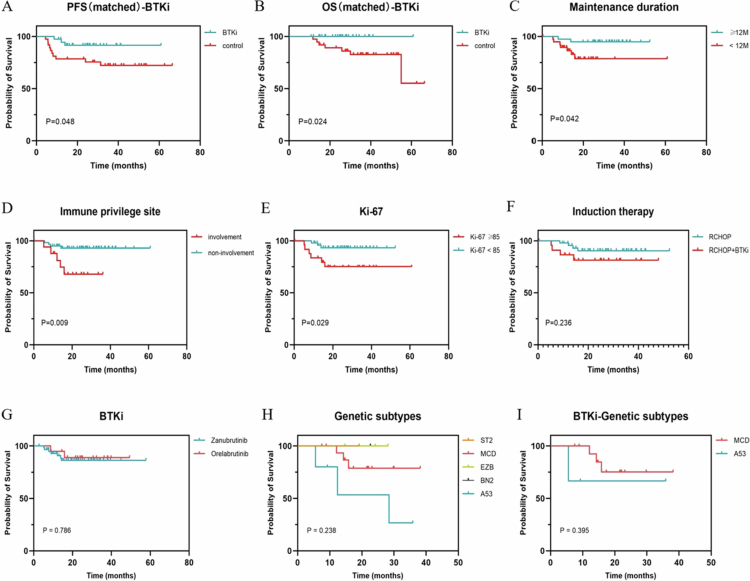
Subgroup analysis of maintenance therapy with BTKi. (A and B) PFS (A) and OS (B) of BTKi maintenance therapy versus no maintenance therapy (REMoDL-B cohort). (C–I) These figures are only analyzing the patients who received BTKi maintenance. PFS survival curves of duration of maintenance therapy (C), immune privilege site involvement or not (D), Ki-67 level (E) and induction regimen (F) in patients receiving BTKi maintenance therapy. (G) PFS survival curves for two different BTKi. (H) PFS survival curves of different molecular subtypes in all patients receiving maintenance therapy. (I) PFS curves of MCD and A53 in patients receiving BTKi maintenance therapy. PFS progression-free survival, OS overall survival, BTKi Bruton tyrosine kinase inhibitors.

In this study, MT included BTKi, rituximab, and lenalidomide (Table 2). The choice of drug is mainly made by physicians to screen the population likely to benefit based on published studies. In addition, financial effects and patient adherence were taken into account. Of these, 73.58% (78/106) of patients received BTKi as MT. The 2-y PFS and OS rates were 87.6% and 97.2%, respectively. Among patients classified as high-risk according to the IPI (IPI score ≥ 3) and the CNS-IPI (CNS-IPI score ≥ 4), the 2-y PFS rates were 84.4% and 82.6%, respectively. All patients had no CNS involvement confirmed by PET-CT/brain MRI and cerebrospinal fluid examination at baseline. However, in 8 patients, the tumors were located in the sinuses/orbit and involved the skull base. Notably, 43.6% (34/78) of the patients were categorized as high-risk based on the CNS-IPI. 20.5% (16/78) of the patients were considered to have high-risk factors for central progression/recurrence due to the involvement of special sites (bone marrow, uterus, breast, testis, skull base). Due to safety concerns, only 44% (22/50) of high-risk patients younger than 70 y with normal creatinine levels were given 2 cycles of intravenous methotrexate as central prophylaxis. Importantly, no CNS recurrences were observed during the follow-up period. Through PSM, 37 patients with DLBCL from the database were selected to form the control group (Table 1). The BTKi maintenance group demonstrated significantly improved PFS and OS compared to the control group (*p* = 0.048 and *p* = 0.024, respectively; Figure 4A and B). Patients who received MT for a duration of one year or more exhibited higher PFS than those who received less than one year of maintenance (*p* = 0.042, Figure 4C). However, PFS was adversely affected when the disease involved immune-privileged sites (*p* = 0.009) and when Ki-67 expression was ≥85% (*p* = 0.029) in the BTKi maintenance group (Figure 4D and E). The estimated 2-y PFS rates for patients with and without involvement of immune-privileged sites were 67.9% and 93.1%, respectively. The incorporation of BTKi during induction therapy did not result in significant PFS benefits (*p* = 0.236, Figure 4F). The decision to undergo ASCT did not significantly impact the PFS of patients on BTKi maintenance therapy (*p* = 0.805). Furthermore, no significant differences in PFS were observed between the zanubrutinib and orelabrutinib (*p* = 0.786, Figure 4G). Patients with the MCD/A53 molecular subtype exhibited poorer PFS outcomes (Figure 4H), with those receiving BTKi maintenance therapy showing worse PFS in the A53 subtype compared to the MCD subtype (Figure 4I).

**Table 2.** The maintenance therapy regimens.

Agents	BTKi (*n* = 78)	Rituximab (*n* = 26)	Lenalidomide (*n* = 2)
Dosing	Zanubrutinib (160 mg bid)/ Orelabrutinib (150 mg qd)	375 mg/m^2^ every 3 months	20 mg/d1–21 of 28 d
Start time	Confirmed response at EOT		
Intended duration	2 y		
Stopping rules	Adverse events reached the criteria for permanent discontinuation or patients requested withdrawal		
Median duration	11.5 months	12 months	7 months

BTKi – Bruton tyrosine kinase inhibitors, EOT – end of induction therapy.

### Safety

The primary hematological adverse reaction during BTKi maintenance was thrombocytopenia, with an incidence of grade 3 or higher thrombocytopenia at 2.56% (2/78). Non-hematological adverse reactions included infection (9/78), abnormal liver function (1/78), atrial premature beats (1/78), non-infectious fever (1/78), and cerebral infarction (1/78). Notably, no grade III or higher non-hematological adverse events were reported. These adverse events were manageable through dose reduction or temporary discontinuation of the medication.

## Discussion

This study presents a real-world, multicenter retrospective analysis of MT in DLBCL, offering contemporary insights in the context of novel therapeutic agents. It is also the first study to directly compare multiple maintenance strategies. Our findings demonstrate that PFS and OS were significantly higher in patients receiving MT compared to those who did not. This observation appears to contradict previous evidence. A 2019 meta-analysis by Rozental et al. suggested that MT reduced relapse rates in DLBCL but did not improve OS.^13^ Similarly, a 2021 meta-analysis by Yuan et al. reported no OS benefit from MT for DLBCL patients.^14^ It is noteworthy that the maintenance regimens in these studies primarily consisted of rituximab and lenalidomide. Importantly, data on BTKi as MT were lacking. Furthermore, the induction regimens in these earlier studies were predominantly R-CHOP-like, as BTKi had not yet been incorporated into first-line treatment. These differences may account for the divergent results observed in our study. This underscores how the continuous emergence of new agents may challenge conventional treatment paradigms previously supported by older data.

It is important to highlight that clinicians in real-world settings tend to favor administering MT to patients identified as high-risk. In our MT cohort, there was a higher prevalence of patients exhibiting adverse prognostic factors, such as age greater than 60 y, ECOG performance status of 2 or higher, stage III-IV, elevated LDH levels, involvement of more than one extranodal site, an IPI score of 3 or greater, classification within the high-risk CNS-IPI group, and involvement of immune-privileged sites. It is well-documented that the 5-y OS rates for IPI low, low-intermediate, high-intermediate, and high-risk groups are approximately 73%, 51%, 43%, and 26%, respectively.^15^ Regardless of prognostic risk, the 2-y PFS rates following R-CHOP and R-CHOP-like induction therapies range from 62.8% to 84.1%.^16^ The 2-y PFS rate with MT was approximately 72% to 88.5%.^16^ In our study, the 2-y PFS rate reached 90%. Notably, even within the subgroup characterized by predominantly poor prognostic indicators, MT resulted in a PFS rate surpassing previously reported data and comparable to the highest PFS rates documented for MT. Furthermore, within the IPI high-risk group, the 2-y PFS rate was as high as 87%. Meanwhile, among patients undergoing MT, PFS did not differ between the high-risk and low-risk groups as defined by the IPI and the CNS-IPI. These findings suggest that MT may enhance the prognosis of patients with DLBCL, particularly those with high-risk profiles. Furthermore, our study revealed that patients who achieved only PR at the EOT but subsequently received MT exhibited PFS outcomes comparable to those who achieved CR. This observation implies that MT may enhance the depth of response in patients with PR. It was observed that the majority of patients achieving only PR were elderly, potentially due to their general health status and limited tolerance, which constrained the intensity of the induction therapy. These patients entered MT because clinicians assessed that they would not tolerate intensive chemotherapy and because of the lack of additional targeted options until 2024. Our findings indicate that MT could potentially compensate for suboptimal chemotherapy dosing, which may otherwise result in insufficient remission depth and hinder long-term survival, particularly in elderly patients. Additionally, a maintenance duration of at least one year was correlated with improved PFS, suggesting that MT should be sustained for a minimum of one year.

Regrettably, our research indicates that patients undergoing MT who present with involvement of immune-privileged sites and a Ki-67 index of ≥85% continue to experience poorer PFS. This observation aligns with the clinical characteristic of a high recurrence rate observed in DLBCL affecting immune-privileged sites. The prognosis for DLBCL in these sites is notably worse compared to DLBCL with intracranial involvement, largely due to the high genetic heterogeneity and unique tumor microenvironment within immune-isolated areas, which render the tumor cells less susceptible to anti-tumor immune responses from T cells and natural killer cells.^17^ Furthermore, the presence of the blood-brain barrier and blood-testis barrier complicates treatment efforts.^17^ Our findings suggest that the MT regimens currently in widespread use do not adequately address the clinical requirements for improving outcomes in more aggressive and heterogeneous forms of DLBCL. Consistent with prevailing guidelines, consideration of ASCT or radiotherapy consolidation therapy is recommended for DLBCL in immune-privileged sites.^17^ Future research should focus on the development of more effective pharmacological agents and therapeutic strategies to enhance the prognosis for these patients.

The selection of an optimal maintenance agent continues to pose a significant clinical challenge. Beyond the established agents rituximab and lenalidomide, the Phase III PILLAR-2 trial demonstrated that the mTOR inhibitor everolimus did not enhance survival outcomes as a MT.^18^ Similarly, the Phase III PRELUDE trial found no survival benefit associated with the use of the protein kinase C-β inhibitor enzastaurin as a MT in patients with DLBCL.^19^ The PD-1 antibody avelumab was evaluated as both an induction and MT in a Phase II study, which yielded promising overall response rates; however, additional survival data are necessary.^20^ In our study, we identified for the first time that BTKi as MT can improve PFS and OS, thereby confirming the efficacy of BTKi as a maintenance strategy. The two commonly used BTKis, zanubrutinib and orelabrutinib, exhibited no significant differences in outcomes. The 2-y PFS rates for the IPI high-risk group and the CNS-IPI high-risk group were as high as 84.4% and 82.6%, respectively. The 2-y CNS progression rates for the CNS-IPI low, intermediate, and high-risk groups were 0.6%, 3.4%, and 10.2%, respectively.^21^ Our study included more than half of patients at high-risk of central progression/relapse as classified by CNS-IPI or specific site involvement. However, only 44% of these patients received central prophylaxis. During a median follow-up period exceeding two years, no CNS recurrence was observed in patients who received BTKi as MT, suggesting the promising efficacy of BTKi in preventing CNS recurrence. For patients undergoing BTKi maintenance, the earlier integration of BTKi during induction therapy did not influence PFS. This indicates that the omission of BTKi in the initial treatment regimen does not diminish its beneficial impact on PFS when used solely as maintenance therapy. It is also recommended that the duration of MT be at least one year. The performance of ASCT did not appear to affect the PFS of patients receiving BTKi as MT. This finding is particularly significant for patients who are unsuitable candidates for ASCT, such as the elderly. In cases involving immune-privileged sites, the 2-y PFS for CNS DLBCL is typically no more than 50%.^22^^,^^23^ In our study, the estimated 2-y PFS rate for DLBCL with immune-privileged site involvement treated with BTKi maintenance was 67.9%, which is slightly higher than previously reported data. However, a substantial disparity in PFS remains when compared to patients without immune-privileged site involvement. Addressing the improvement of prognosis for this patient cohort remains a critical challenge.

Within the framework of the “seven-gene” genotyping model, the 5-y OS rates for the MCD, A53, BN2, and ST2 subtypes are reported as 40%, 63%, 67%, and 84%, respectively.^12^ Our study corroborates that, despite maintenance therapy, the MCD and A53 subtypes continue to exhibit the poorest prognoses. In our cohort, these subtypes predominantly received BTKi maintenance, with the MCD subtype demonstrating a trend towards improved outcomes compared to A53. Although this trend did not reach statistical significance, it implies that BTKi maintenance may be more efficacious for the MCD subtype, whereas current therapeutic strategies remain insufficient for enhancing outcomes in the A53 subtype.

Our research indicates that BTKi as MT is both safe and manageable, with common adverse events including thrombocytopenia and infection. These adverse events were reversible upon dosage reduction or temporary cessation of the medication.

Study limitations: (1) This is a retrospective study with a small sample size and short follow-up time; (2) Because almost all high-risk patients were treated with maintenance therapy in the participating centers, this study used an external control cohort. Despite the matching of baseline characteristics, there were still some unmeasured confounding factors; (3) Differences in induction therapy may lead to differences in the depth of remission and affect the outcome; (4) There is a lack of analysis of more factors that may affect prognosis, including double/triple hit, CD5+, and so on. In the future, prospective randomized controlled trials are expected to validate the role of MT.

## Conclusion

In conclusion, the data suggest that MT may be associated with enhanced outcomes in DLBCL in the novel agent era. The findings indicate a significant benefit in PFS and OS, particularly for patients with adverse prognostic indicators such as the high risk of IPI or high risk of CNS-IPI. MT facilitates patients who achieve PR at the EOT to attain survival outcomes comparable to those achieving CR. Consequently, we hope for a more proactive consideration of MT for high-risk DLBCL patients and those achieving only PR, with a duration of at least one year. Furthermore, BTKi maintenance may be an appropriate option for patients at high risk of CNS relapse and those with molecular subtypes of MCD. However, this study is subject to limitations, including a small sample size, a relatively short follow-up period, and its retrospective nature. Future prospective studies involving larger cohorts are necessary to assess a broader spectrum of maintenance strategies.

## Supplementary Material

Supplementary material STROBE checklist.docx

## Data Availability

The datasets generated during and/or analyzed during the current study are available from the corresponding author on reasonable request.
